# Emerging monkeypox cases amid the ongoing COVID-19 pandemic in the Indian subcontinent: A probable healthcare challenge for South East Asia

**DOI:** 10.3389/fpubh.2022.1066425

**Published:** 2022-11-25

**Authors:** Ranjan K. Mohapatra, Snehasish Mishra, Venkataramana Kandi, Ashish K. Sarangi, Md. Kudrat-E-Zahan, Md. Sajid Ali, Rudra Narayan Sahoo, Nawazish Alam, Gurudutta Pattnaik, Kuldeep Dhama

**Affiliations:** ^1^Department of Chemistry, Government College of Engineering, Keonjhar, Odisha, India; ^2^School of Biotechnology, KIIT Deemed to be University, Bhubaneswar, Odisha, India; ^3^Department of Microbiology, Prathima Institute of Medical Sciences, Karimnagar, Telangana, India; ^4^Department of Chemistry, School of Applied Sciences, Centurion University of Technology and Management, Bhubaneswar, Odisha, India; ^5^Department of Chemistry, Rajshahi University, Rajshahi, Bangladesh; ^6^Department of Pharmaceutics, College of Pharmacy, Jazan University, Jazan, Saudi Arabia; ^7^School of Pharmacy and Life Sciences, Centurion University of Technology and Management, Bhubaneswar, Odisha, India; ^8^Department of Pharmacy Practice, College of Pharmacy, Jazan University, Jazan, Saudi Arabia; ^9^Division of Pathology, ICAR-Indian Veterinary Research Institute, Bareilly, Uttar Pradesh, India

**Keywords:** monkeypox disease, public health, treatment, prevention and control, South East Asia

The recent 2022 monkeypox outbreak poses an emergency international public health concern amid rising cases as an all-time biggest outbreak (1). Monkeypox (MPX) was reported in some African countries initially before its 2022 outbreak. While some African countries had persistent or recurrent outbreaks, some others had discrete cases. Countries having travel links with Nigeria have also reported cases occasionally. MPX spreads to humans through close contact with infected rodents and primates. Individuals who either live with or have close or regular contact (e.g., skin-to-skin, mouth-to-skin, face-to-face, or mouth-to-mouth) with MPX-infected animals are at greater risk. Healthcare workers are recommended to self-protect by following infection prevention and control measures while dealing with MPX patients. To prevent reverse infection, humans with confirmed or suspected MPX need to avoid close animal contact with pets (dog, cat, hamster, gerbil, etc.), livestock, and wildlife (2). They need to be particularly vigilant about rodents and non-human primates in their surroundings, the known MPX-susceptibles.

MPX virus is a double-stranded DNA virus of the Orthopoxvirus genus and Poxviridae family, of two clades, *viz*., Congo Basin (Central African) clade and the West African clade (3). Studies suggest that human infections by the West African clade are less severe than the Congo Basin's, and the fatality rate in the former is 3.6% compared to 10.6% in the latter. The natural history, animal origin, and reservoir host of MPX are yet to be ascertained. For a better understanding of the zoonotic origin of the MPX virus in endemic and non-endemic areas is highly essential through intensive surveillance. Developing a surveillance system to gather information and identify the knowledge gaps in MPX research is also important to develop a due and diligent action plan for prevention and control.

It is still unclear whether MPX spreads through semen or vaginal fluids although an MPX patient is advised to use condoms for 12 weeks after recovery and until the semen tests negative. MPX spreads through other close contacts, possibly through sexual contact, with one who is MPX infected. Although it might help protect from other STIs, a condom may allegedly not protect from MPX. Children in close contact with MPX patients are also infected, and in fact, they are typically more prone to severity than adolescents and adults. As there have been few cases of MPX-infected children in the current outbreak, more cases if any could validate this. Studies to understand the risks of MPX during pregnancy and whether the virus transmits vertically either during parturition or during breastfeeding are also needed. Available data suggest that contracting MPX during pregnancy could put the fetus at risk (2).

“Tecovirimat,” an antiviral developed to treat smallpox is now approved to treat MPX. Other antivirals like cidofovir, brincidofovir, and vaccinia immune globulin intravenous (VIGIV) may also be useful to treat critical MPX cases such as in pregnant or breastfeeding women, pediatrics, or the immunocompromised (3–6). The understanding of how best their use shall benefit will improve as data accumulate in the future. The symptoms resolve without the need to treat which is a reason for respite. To minimize fluid loss and for relief, along with other supportive care medication for pain (analgesics) and fever (antipyretics) may be used. The rash may be cleaned with sterile water or antiseptic, and for mouth lesions, saline water rinsing may be resorted to. A warm bath with baking soda and Epsom salts may help against body lesions, and Lidocaine could be used against oral and perianal lesions (2). As the African healthcare system is already struggling due to the multiple waves of the COVID-19 pandemic and the emerging SARS-CoV-2 variants, governments could implement numerous prescribed preventative measures to prevent its spread. Healthcare workers must be amply conversant about the presenting signs and symptoms, and the suspected patients may be quarantined promptly while receiving symptomatic treatments (7).

The current 2022 MPX outbreak in many countries at once is atypical to previous instances. MPX reportedly spread among the MSM in the non-endemic regions through close contact like touching, kissing, and oral and penetrative vaginal or anal sex with infectious individuals (3). The rash may develop in hard-to-notice regions like the throat, mouth, genitals, vagina, and anus/anal area. The general public of non-endemic countries like India is MPX infected, as per recent reports. MPX, it seems, is not only restricted to the MSM group but across all groups as a global threat. Currently, the population at risk is only considered for vaccination, and not mass vaccination (2). The smallpox-vaccinated individuals may be protected against MPX to a certain extent. As smallpox vaccination stopped worldwide after its eradication in the 1980's, the young population is not smallpox vaccinated which is worrisome for MPX infection. Past exposure to the Varicella virus causing chickenpox does not protect against MPX (2). Studies to understand how people are exposed to MPX, and actions to establish public health interventions to limit the further spread are underway.

When the WHO declared MPX a ‘public health emergency of international concern' in June 2022, some 3,000 cases were identified in nearly 50 countries. More than 75,000 cases across 109 countries and territories are reported as on October 21, 2022 (8). Importantly, 74,457 cases were identified from 102 countries and territories including the ones with no reported MPX history, compared to only 891 cases reported from seven endemic countries with a history (8). Non-endemic countries like the USA (27884), Brazil (8860), Spain (7277), France (4084), the UK (3686), Germany (3656), Colombia (3110), Peru (2913), and Mexico (2468) have reported large cases as on October 21, 2022 (8). That MPX cases are rising in non-endemic countries across economic strata is a matter of great health concern. It is a rare but potentially serious viral illness. Previous outbreaks were limited to the African subcontinent, mainly the Democratic Republic of the Congo (163) and Nigeria (133), with a few cases from Ghana (30), the Central African Republic (08), Cameroon (07), and Liberia (01).

Recently, India reported discrete MPX cases from districts of Kerala and Delhi. So far, India has reported 14 MPX confirmed cases along with some suspected cases in a relatively short time. These are the first reported cases by the WHO in South-East Asia (9). To date 21 MPX-related deaths (including one from India) are reported from countries and/or territories with no MPX history. The issue affects the Indian subcontinent in particular and South-East Asia in general. The south-East Asian region is the second most populated region in Asia with a population density of 154/Km^2^, comprising mainland South-East Asia (Indochinese Peninsula; Laos, Cambodia, Myanmar, Thailand, Vietnam, Peninsular Malaysia), and maritime South-East Asia (Malay Archipelago; East Malaysia, Brunei, East Timor, Philippines, Indonesia, and Singapore). For various reasons, from a pandemic viewpoint, South-East Asia is a delicate and sensitive region. These reasons include its population density and the overall percentage of the global population, the extremely porous international borders for anthropogenic and economic activities, global reach being well-connected to the world by air, and the outward spread of the population (being found almost in every nook-and-corner of the globe).

Earlier, 75 suspected MPX-related deaths were reported in Africa, mostly in Nigeria and the Congo, supposed to be more lethal MPX than in the West (2). As per data, MPX is less fatal compared to the Variola virus. Although MPX is clinically less severe compared to smallpox, the symptoms are nearly similar. With the eradication of smallpox and subsequent cessation of its vaccination, MPX emerges as a critical orthopoxvirus. In this scenario, the newborn, young children, and immunocompromised individuals are at risk of fatal infection. B.1 is responsible for the current 2022 MPX outbreak although many virus variants and lineages (A, A.1, A.1.1, A.2, and B.1) have been identified to date (10).

As per a recent ICMR study, the retrieved MPXV sequences from India belong to A.2 lineage. The MPXV strains sequenced from Indian cases revealed that the virus had undergone significant genetic changes as noted by APOBEC3 gene mutation and an additional 16 SNPs (11). Thus, this variant differs from the B.1 variant that is prevalent in European countries and the A.1 strain that was found during 2017–18 outbreaks in Nigeria, Singapore, and Israel. The MPXV variants in Indian cases varied from the currently prevalent ones in the UK (UK-2022 OP331335.1) and the US (USA-2022 ON674051.1). Identifying and naming MPXV variants in the future based on their similarity or in line with the existing variants is imperative. It was the reason why Indian cases were attributed to a new A.2.1 sublineage. Revised nomenclature of MPXV variants suggests human MPXV basic lineage be considered as A, and the sublineages are named as A.1, A.2, A.1.1, etc., and B.1 as the first variant from A.1.1 sublineage. Isidro et al. (12) suggested that the ongoing 2022 outbreak may be attributed to the virus that emerged from a single MPXV clade and that the B.1 could have emerged from A.1 itself.

A recent report indicated an alarming upsurge in diarrhea cases in Bangladesh which makes the case worse in this South-East Asian country amid the ongoing COVID-19 pandemic. About 4.5 million across the country are diarrhea infected in the first 3 months of the year (13). Healthcare systems in South-East Asia are overburdened due to the emerging SARS-CoV-2 variants along with other frequently reported diseases (14). The WHO has officially declared a number of deaths by the highly infectious, Ebola-like Marburg virus in Ghana amid the ongoing COVID-19 pandemic (15). This outbreak is the second reported outbreak in West Africa after the Guinea outbreak last year. As per WHO, Ebola cases due to the Zaire virus and Sudan virus is also detected recently this year in the African continent. Africa documented an increase in Ebola, Marburg, and other infectious animal-human diseases and their further transmission among humans (16). Like the MPX, they may affect adversely if they enter other countries. Healthcare surveillance systems need to be more vigilant and bolster their preparedness, disease scrutiny, and the swift detection of cases. Such viral zoonoses, their management, and treatment lack attention, being neglected diseases. Research for effective antiviral drugs, immunotherapy and supportive therapies, and other advanced interventions are highly essential and need to be given due priority.

Amid the rising MPX cases in India, the Serum Institute of India (SII) is set to manufacture a vaccine against it. Currently, the licensed vaccine for MPX is of a Danish firm, and the live virus on which it is based is stored at two locations, at the State Research Center of Virology and Biotechnology Institute, Koltsovo, Russia, and the Center for Disease Control, Atlanta, Georgia. The National Institute of Virology (NIV, Pune) reportedly has been successful in isolating the MPX virus from a patient sample that shall help develop a vaccine and test kits (https://www.livemint.com/news/india/monkeypox-8-cases-in-india-so-far-1-death-10-things-to-know-11659430193160.html). The COVID-19 mistake must not be repeated lest the world should pay the price for having remained inadequately responsive (17). Since such diseases are ignored for decades, preventives and therapeutics are not globally available. High-income nations must learn from the mistakes committed during the COVID-19 pandemic and should support through emergency vaccine shots, diagnostics, and treatments, with effective targeting. As per the US Agency for International Development (USAID), low-income countries failed in the COVID-19 response (17). Vaccine donors and vendors need to collaborate with researchers and healthcare systems to determine the requirements of each country to scale up the response to infectious diseases, particularly the potentially pandemic ones. Collective regional and global partnerships are imminent to strengthen MPX preparedness and implement the action plans. Understanding the human–animal–environment interface and transmission dynamics with a regional and global ‘One Health' approach is suggested (18).

## Author contributions

RKM designed the study and made the first draft. MA, RNS, and AKS teamed up during the first draft. SM, VK, MK-E-Z, and NA updated the manuscript and edited it. KD and GP reviewed the final draft. All authors have critically reviewed and approved the final draft, and are responsible for the content and similarity index of the manuscript.

## Conflict of interest

The authors declare that the research was conducted in the absence of any commercial or financial relationships that could be construed as a potential conflict of interest.

## Publisher's note

All claims expressed in this article are solely those of the authors and do not necessarily represent those of their affiliated organizations, or those of the publisher, the editors and the reviewers. Any product that may be evaluated in this article, or claim that may be made by its manufacturer, is not guaranteed or endorsed by the publisher.
